# Compressive myelopathy and compression fracture of aggressive vertebral hemangioma after parturition: A case report and review of literature

**DOI:** 10.1097/MD.0000000000018285

**Published:** 2019-12-16

**Authors:** Guan-xi Wang, Yuan-dong Mu, Jun-yi Che, Guang-fei Zhang, Gang Jiang, Chuan-ping Gao

**Affiliations:** a Department of Radiology, Songshan Hospital of Qingdao University Medical College University; b Department of Radiology, Gaomi people Hospital, Weifang; c Department of Radiology, Qingdao Municipal Hospital; d Department of Radiology; e Department of Radiology; f Department of Radiology, The Affiliated Hospital of Qingdao University, Qingdao, China.

**Keywords:** aggressive vertebral hemangioma, compression fracture, compressive myelopathy, MRI, PET-CT, post-parturition

## Abstract

**Rationale:**

Compressive myelopathy and compression fracture of aggressive vertebral hemangioma after parturition is a rare condition. Vertebral body compression fracture and high serum progesterone lead to extraosseous hemangioma enlargment cause narrowing the spinal canal which contribute to compressive myelopathy relate to pregnancy.

**Patient concerns:**

We report a case of compressive myelopathy and compression fracture of aggressive vertebral hemangioma after parturition in a 35-year-old woman. The patient complained unable to walk and experienced intense pain in the back.

**Diagnosis:**

Based on the clinical features and imaging studies, the patient underwent a T4–T6 laminectomy. Histopathology consistent with vertebral hemangioma.

**Interventions:**

The patient underwent laminectomy for decompression. After subperiosteal dissection of the paraspinal muscles and exposure of the laminae, there was no involvement of the lamina by the tumor. The epidural tumor was removed through the spaces lateral to the thecal sac. Vertebroplasty was performed through T5 pedicles bilaterally and 7 ml of polymethylmethacrylate (PMMA) cement was injected. T4–T6 pedicle screw fixation was performed for segmental fixation and fusion.

**Outcomes:**

Six months after resection of the tumor the patient remained asymptomatic. She reported no low back pain and had returned to her normal daily activities, with no radiographic evidence of recurrence on MRI. Physical examination revealed that superficial and deep sensation was restored to normal levels in the lower extremities.

**Lessons:**

The occurrence of compressive myelopathy of pregnancy related vertebral hemangiomas is quite unusual. It can lead to serious neurologic deficits if not treated immediately. So, prompt diagnosis is important in planning optimal therapy and preventing morbidity for patients.

## 1 Introduction

Vertebral hemangiomas are the most common benign tumors of the spine and have a prevalence of 10% to 12% of the general population.^[1]^ Aggressive vertebral hemangiomas may extend into the par spinal and epidural spaces. Pregnancy can exacerbate the growth of hemangiomas, resulting in spinal cord compression.^[2–6]^ Compressive myelopathy related to pregnancies of vertebral hemangiomas may occurred during gestation or postpartum and there were 33 cases had reported in the literatures. Most common clinical presentation of reported patients were paraplegia. We herein report a rare case of an aggressive vertebral hemangioma that caused a compression fracture and compressive myelopathy 4 days after vaginal delivery. There were 6 cases aggressive vertebral hemangioma caused compressive myelopathy post-parturition and the patient we reported was the first case that occurred after delivered the second baby. The current case was aggressive vertebral hemangiomas and the component extended to epidural space shows no uptake in PET-CT which may differentiated from neurinoma, meningioma, and lymphoma. We also reviewed literatures and analyzed compressive myelopathy related to pregnancies.

## 2 Case report

A 35-year-old woman with no relevant medical history was admitted to our hospital with a 15-day history of progressive numbness and weakness of her bilateral lower extremities that began 4 days after vaginal delivery. She had given birth to a child 5 years before and the history was unremarkable. After admitted, the patient was unable to walk and experienced intense pain in the back, especially during mobilization. Physical examination showed percussion pain at the T4 to T8 level. Neurological examination revealed decreased muscle strength in the bilateral lower limbs. Her muscle strength was grade 3 in both lower extremities. Sensory examination showed impaired temperature and pain sensations with a sensory level at T6. The Babinski sign was positive bilaterally.

**Figure 1:**
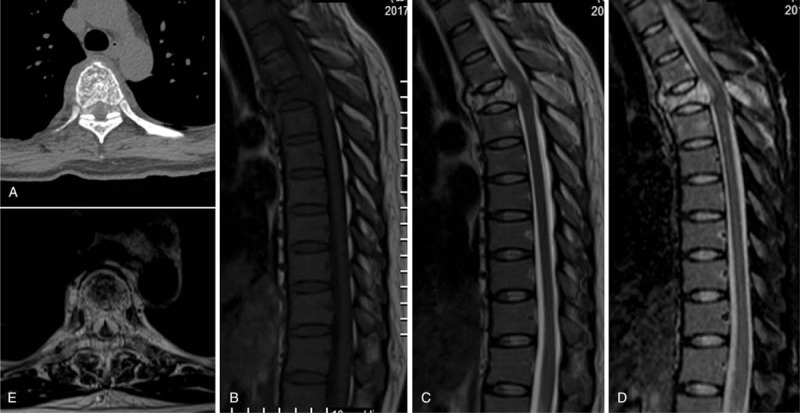
(A) Computed tomography shows sclerosis of the cancellous bone and a fracture involving the anterior cortex of the vertebral body.(B) Magnetic resonance imaging (MRI) showsa compression fracture of the T5 vertebra.(C) MRI shows T5 vertebral body compression in the dural sac and spinal cord.(D) MRI showsahemangioma of the T5 vertebral body and hyperintensity of the spinous process. (E) MRI shows an extraosseous component of the hemangioma (arrow), indicating an aggressive vertebral hemangioma.

**Figure 2:**
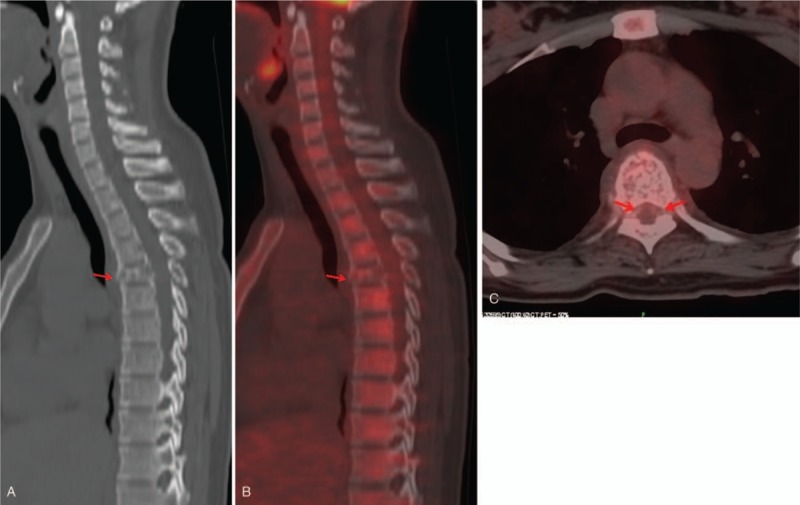
(A) Computed tomography (CT) shows thickenedvertebral trabeculae within theT5 vertebral body.(B) Positron emission tomography–CT (PET-CT) shows milduptakein the T5 vertebral body.(C) PET-CT shows no uptake in the extraosseous components of the lesion.

Computed tomography (CT) showed sclerosis of the cancellous bone involving the entire T5 vertebra with epidural spaces extension, including the epidural space (Fig. 1A). Conventional MRI revealed a compression fracture at T5 with anterior compression of the spinal cord (Fig. 1B–E). 18-Fluorodeoxyglucose (FDG) positron emission tomography–CT (PET/CT) demonstrated compression fracture and mild uptake in the T5 vertebral body (Fig. 2A-C) with a maximum standardized uptake value of 3.2.

**Figure 3:**
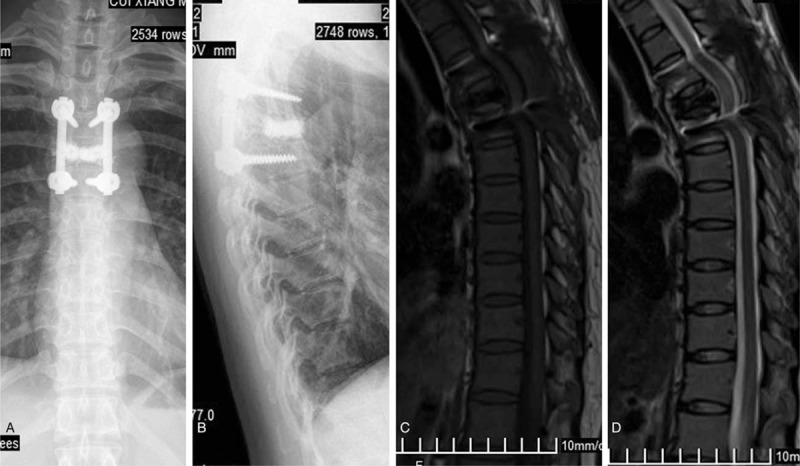
Postoperative anteroposterior (A) and lateral (B) radiographs demonstrating spinal fixation. The T5 vertebral body was filled with methylmethacrylate. T1WI (C) and T2WI (D) show decompression of the spinal cord in 6-month follow-up after surgery.

Based on the clinical features and imaging studies, the patient underwent a T4–T6 laminectomy. After subperiosteal dissection of the paraspinal muscles and exposure of the laminae, there was no involvement of the lamina by the tumor. A firm epidural mass was found filling the anterior epidural space and compressing the cord. The epidural tumor was removed through the spaces lateral to the thecal sac. The dura was found to be normal with no evidence of infiltration from the tumor. Vertebroplasty was performed through T5 pedicles bilaterally and 7 ml of polymethylmethacrylate (PMMA) cement was injected (Fig. 3A-B). T4–T6 pedicle screw fixation was performed for segmental fixation and fusion (Fig. 3 A-D).

Her symptoms were rapidly relieved after the surgery. Postoperative plain x-ray showed a good alignment of the thoracic spine and stable fusion of T4-T6. Six months after resection of the tumor the patient remained asymptomatic, with no clinical or radiographic evidence of recurrence on MRI (Fig. 3C-D). Physical examination revealed restoration of sensory function, as well as improved motor function (muscle strength 3/5) in her lower extremities.

## 3 Discussion

Vertebral hemangiomas are common benign vascular tumors. The most frequent location of these lesions is within the lower thoracic and lumbar vertebrae, and they are often multiple.^[7–9]^ The typical radiological characteristic of vertebral hemangiomas is vertical striation sproduced by zones of reduced bone density between more dense trabeculae.^[1,8,10]^ The lesion may involve the pedicles, arches, and spinous processes. The vast majority of these lesions are asymptomatic.^[11]^

The term “aggressive hemangioma” refers to a vertebral hemangioma with extraosseous extension.^[2]^ The affected vertebra is usually located between T3 and T8.^[3]^ Vertebral hemangiomas can cause neurologic symptoms by means of various mechanisms. For example, enlargement of the vertebral body leads to narrowing and distortion of the spinal canal and a symptomatic compression fracture.^[4]^

Compression fracture of an involved vertebra is infrequent because the hemangiomatous vertebra is reinforced by thick sclerotic vertical trabeculae from new bone formation.^[5]^ Pathophysiologically, vertebral hemangiomas cause diffuse bone infiltration and consequently reduced bone density. The lesions are characterized by erosion of the horizontal trabeculae. Rarely, their enlargement within the vertebral body is weakened by the hemangioma. These 2factors are responsible for pathological fracture under axial loading.^[6]^

There were 33 cases vertebral hemangiomas, including present case, with compressive myelopathy related to pregnancies reported in the literatures which listed in Table 1. Among those reported, 21 of the cases with extraosseous extension which called aggressive hemangioma. Furthermore, 1case with intrdural angioma from T11 to L3 level, and 1 case with intraspinal bleeding.

**Table 1:**
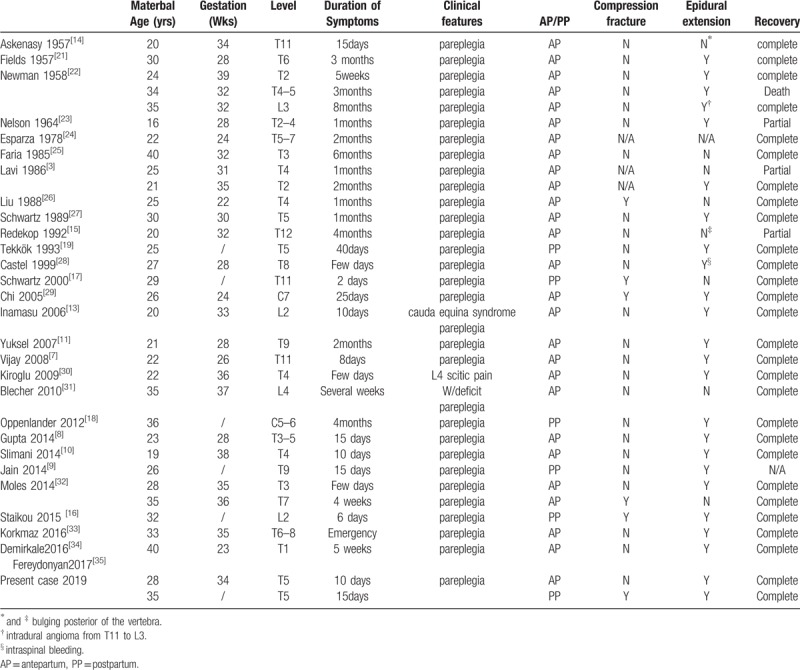
Previously published cases of pregnancy-related vertebral hemangioma.

Pregnancy is known to provoke progression of a vertebral hemangioma by inducing a 30% to 50% increase in blood volume. The 2 main mechanisms for dilatation or expansion of hemangiomas during pregnancy are as follows:

1. the gravid uterus may compress the inferior vena cava, resulting in a venous pressure increasein the paravertebral venous plexus, and
2. hormonal changes such as high serum progesterone may lead to expansion of the hemangioma and development of neurological symptoms.^[3,12]^

In this condition, mechanical factors such asbending and axial vertebral loading lead to the development of a pathological compression fracture.^[13]^ In the reported compressive myelopathy related to pregnancies, 2 case caused compressive myelopathy because of bulging of the vertebrae which reported by Askenasy et al^[14]^ and Redekop et al^[15]^ respectively,6 cases with vertebral compression fracture. Among these 6 patients, 2 cases were aggressive hemangioma which may aggravate clinical symptom.

Askenasy et al^[14]^ reported the first case of a symptomatic vertebral hemangioma during pregnancy in 1957. Among the reported 33 case compressive myelopathy related to pregnancies, 6 cases were post parturition. The present case is unusual because neurological deterioration did not begin until after parturition, and her symptom occurred 4 days after vaginal delivery. Staikou et al^[16]^ and Schwartz et al^[17]^ reported aggressive vertebral hemangiomas with compression fractures with immediate development of compressive myelopathy after vaginal delivery, and the imaging findings are similar to present case. Two cases of compressive myelopathy without compression fracture were reported by Oppenlander et al^[18]^ and Tekkök et al,^[19]^ respectively. The specific time at which the myelopathy occurred in the report by Tekkök et al^[19]^ was unavailable, unlike present case, extradural component of hemangioma located at the posterior aspect of the spinal cord. The myelopathy reported by Oppenlander et al^[18]^ occurred 4 months after the patient gave birth, in this patient, extradural component of the hemangioma show big mass which located intervertebral foramen and paraspinal space.

The characteristic MRI appearance of a vertebral hemangioma involves T1 and T2 hyperintensity on non-contrast imaging. The extraosseous component is hypointense relative to the marrow on T1-weighted imaging and hyperintense on T2-weighted imaging and shows uniform enhancement on post-enhanced images. 18-FDG PET/CT is helpful for determining the extent of the lesions. The mild uptake in the T5 vertebra may be caused by a compression fracture; however, vertebral hemangiomas without a compression fracture also may show mild FDG uptake. The lack of uptake by an extraosseous component may help to differentiate the lesion from other extradural space-occupying lesions such as hemangioblastoma, schwannoma, ganglioglioma, and metastatic tumors, which usually show moderate FDG uptake.^[20]^

The rapidly increasing size of hemangiomas during pregnancy can cause narrowing of the spinal canal, fracture of the involved vertebral body, causing acute neurological deficit. Patients will warrant emergency surgical decompression.^[16,17]^ In these cases, preoperative embolization procedure is an effective method which can decrease the risk of hemorrhage.^[18]^ Most of the reported 33 patients performed laminectomy and the postoperative course was uneventful with complete neurological recovery.

## 4 Conclusion

The occurrence of compressive myelopathy of pregnancy related vertebral hemangiomas is quite unusual. It can lead to serious neurologic deficits if not treated immediately. So, prompt diagnosis is important in planning optimal therapy and preventing morbidity for patients.^[10]^ The differential diagnosis of aggressive vertebral hemangioma includes nerve sheath tumor, especially extradural component located in the intraspinal space, intervertebral foramen, and paraspinal area, which are dumbbell-shaped. The involved vertebral bodies and elements of hemangioma manifest as thick trabeculae on CT images and the extradural components show homogeneous hyperintense on T2-weighted images, which is the imaging characteristics of aggressive vertebral hemangioma.

## Acknowledgments

We thank Angela Morben, DVM, ELS, from Liwen Bianji, Edanz Editing China (www.liwenbianji.cn/ac), for editing the English text of a draft of this manuscript.

## Author contributions

**Investigation:** Guanxi Wang, Yuandong Mu, Junyi Che.

**Writing – original draft:** Junyi Che, Guangfei Zhang, Gang Jiang.

**Writing – review & editing:** Guanxi Wang, Chuan-ping Gao.
